# Supplementary material for: Orientational Preferences of GPI-Anchored Ly6/uPAR Proteins
Source: Int J Mol Sci. 2022 Dec 20;24(1):11. doi: 10.3390/ijms24010011 (PMC9819746; doi:10.3390/ijms24010011)
Supplement: Supplementary file 1 [file ijms-24-00011-s001.zip › ijms-2072972-supplementary.pdf]

International Journal of Molecular Sciences

MDPI

Supporting information

Orientational Preferences of GPI-anchored Ly6/uPAR Proteins

Maxim M. Zaigraev[1,2], Ekaterina N. Lyukmanova[3,4,*], Alexander S. Paramonov[1], Zakhar O. Shenkarev[1,2], Anton O. Chugunov[1,2,5,*]

[1] Shemyakin-Ovchinnikov Institute of Bioorganic Chemistry Russian Academy of Sciences, Miklukho-Maklaya str. 16/10, 119997, Moscow, Russia
[2] Phystech School of Biological and Medical Physics Moscow Institute of Physics and Technology, Institutskiy per. 9, 141701, Dolgoprudny, Moscow Region, Russia
[3] Faculty of Biology, MSU-BIT Shenzhen University, 518172 Shenzhen, China
[4] Interdisciplinary Scientific and Educational School of Moscow University "Molecular Technologies of the Living Systems and Synthetic Biology", Biological Faculty, Lomonosov Moscow State University, Leninskie Gory, 119234, Moscow, Russia
[5] National Research University Higher School of Economics, Myasnitskaya str. 20, 101000, Moscow, Russia

Correspondence: ekaterina-lyukmanova@yandex.ru (E.N.L.); anton.chugunov@gmail.com (A.O.C)

Supplementary methods

Calculation of protein orientation. To define a protein orientation, the four reference groups (G1, G2, G3, G4) were defined in the protein structure. The COMs of these groups approximately correspond to the positions of head, loop II, loop I, and loop III, respectively. The groups were chosen on the basis of the 3D structure superposition in PyMOL and sequence alignment in Jalview [1] taking into account conservative cysteines *via* CysBar [2] (Fig. S4). COM coordinates of protein backbone for G1, G2, G3, and G4 groups were extracted using *gmx trjcat* procedure. These coordinates were used to define two vectors in conditional three-finger plane of the protein. Vector $\vec{A}$ is directed from G1 to G2 group (from the 'head' of the protein to the tip of the central loop (II) and runs along the conserved antiparallel β-sheet). Vector $\overrightarrow{B'}$ is directed from G3 to G4 group (from the loop I to the loop III). Using vector product we defined vector $\vec{F}=[\vec{A} \times \overrightarrow{B'}]$, orthogonal to the protein three-finger plane, and directed from 'ventral' to 'dorsal' side of the protein β-structure (Figs. S4 and S5A). Next, using vector product again, we defined vector $\vec{B}=[\vec{A} \times \vec{F}]=[\vec{A} \times [\vec{A} \times \overrightarrow{B'}]\ ]$, lying in the three-finger plane, but orthogonal simultaneously to $\vec{A}$ and $\vec{F}$ (Figs. S4 and S5A). Vector $\vec{B}$ is directed approximately from the loop III to the loop I (Fig. S5A). Then using scalar product we calculated two angles: $\varphi=\angle(\vec{A}, \vec{Z})$, $\varphi \in [0°, +180°]$ and $\omega=\angle(\vec{B}, \vec{Z})$, $\omega \in [0°, +180°]$ ($\vec{Z}$ is a normal vector, perpendicular to the membrane, see Figs. S3A and S5A). Based on determined vectors $\vec{A}$, $\vec{B}$, $\vec{F}$ and angles φ and ω we calculated tilt (α) and rotation (β) angles of the protein ultimately defining its orientation in 3D space.

To specify tilt of the protein to the membrane we calculated angle $\alpha = 90° - \varphi = 90° - \angle(\vec{A}, \vec{Z})$, $\alpha \in [-90°, +90°]$ (see Fig. S3B). Tilt angle α is positive, when head (group G1) is lower than the tip of the loop II (group G2) and negative otherwise.

To determine transverse tilt (relative to vector $\vec{A}$) of the molecule (in other words rotation of the protein by loop I, loop III, 'dorsal', or 'ventral' side towards the membrane) we calculated rotation angle β ($\beta \in [-180°, +180°]$). For that we analyzed projections of vectors $\vec{B}$ and $\vec{F}$ on the $\vec{Z}$ axis (B_z and F_z, respectively). Four cases, corresponding to different quadrants (I, II, III and IV) in the Figure S5B, are possible:

I. $B_z \geq 0$ and $F_z \geq 0$: $\beta = 90° - \omega$
II. $B_z \geq 0$ and $F_z < 0$: $\beta = 90° + \omega$
III. $B_z < 0$ and $F_z < 0$: $\beta = -270° + \omega$
IV. $B_z < 0$ and $F_z \geq 0$: $\beta = 90° - \omega$

Using above definitions, the case where the loop I of the protein is oriented towards the membrane corresponds to $\beta = -90°$; the loop III oriented towards the membrane corresponds to $\beta = +90°$; $\beta = 0°$ corresponds to the protein's β-sheet parallel to the membrane and 'ventral' side of the β-structure (containing *N*- and *C*-termini) directed towards the membrane; $\beta = \pm 180°$ corresponds to the case, where 'dorsal' side of the β-structure is directed towards the membrane.

All necessary vector algebra calculations were conducted in Python *via* in-house scripts.

Table S1. RMSD comparison of structures from AlphaFold Database and PDB.

Protein	Uniprot code	Experimental structure (PDB code)	RMSD, Å
**Lynx1**	P0DP58	2L03	0.74
**Lynx2**	Q8N2G4	6ZSS	1.03
**Lypd6**	Q86Y78	6GBI	0.31
**Lypd6B**	Q8NI32	6ZSO	1.52
**Ly6H**	O94772	—	—
**CD59**	P13987	2JB8	0.32

Table S2. Systems of Ly6 proteins for MD study.

<table>
<tr><th>System</th><th>Box dimensions$^{\&}$, nm^3</th><th># of atoms</th><th>MD length, ns</th></tr>
<tr><td>Lynx1/PSM$_{120}$/DOPE$_{48}$/CHL$_{48}$/DOPC$_{24}$/ Water$_{17274}$/Na$^+_{45}$/Cl$^-_{47}$</td><td>7.89×7.89×12.66</td><td>81 556</td><td>3 000</td></tr>
<tr><td>Lynx2/PSM$_{150}$/DOPE$_{60}$/CHL$_{60}$/DOPC$_{30}$/ Water$_{30265}$/Na$^+_{81}$/Cl$^-_{82}$</td><td>8.42×8.42×17.61</td><td>128 239</td><td rowspan="5">2 000</td></tr>
<tr><td>Lypd6/PSM$_{150}$/DOPE$_{60}$/CHL$_{60}$/DOPC$_{30}$/ Water$_{26655}$/Na$^+_{76}$/Cl$^-_{72}$</td><td>8.44×8.44×15.98</td><td>117 861</td></tr>
<tr><td>Lypd6B/PSM$_{150}$/DOPE$_{60}$/CHL$_{60}$/DOPC$_{30}$/ Water$_{23646}$/Na$^+_{63}$/Cl$^-_{64}$</td><td>8.36×8.36×15.01</td><td>108 817</td></tr>
<tr><td>Ly6H/PSM$_{120}$/DOPE$_{48}$/CHL$_{48}$/DOPC$_{24}$/ Water$_{17145}$/Na$^+_{46}$/Cl$^-_{45}$</td><td>7.42×7.42×14.25</td><td>81 636</td></tr>
<tr><td>CD59/CHL$_{144}$/POPC$_{80}$/NSM$_{80}$/POPE$_{16}$/ Water$_{25566}$/Na$^+_{74}$/Cl$^-_{69}$</td><td>8.24×8.24×16.24</td><td>114 061</td></tr>
</table>

$^{\&}$The values of box dimensions for last frames of equilibrium MD are indicated.

Lipids' abbreviations:
POPC — 1-palmitoyl-2-oleoyl-sn-glycero-3-phosphocholine
DOPC — 1,2-dioleoyl-sn-glycero-3-phosphocholine
POPE — 1-palmitoyl-2-oleoyl-sn-glycero-3-phosphoethanolamine
DOPE — 1,2-dioleoyl-sn-glycero-3-phosphoethanolamine
CHL — cholesterol, PSM — palmitoylsphingomyelin, NSM — nervonoylsphingomyelin

The following lipid compositions of membranes were used:
PSM/DOPE/CHL/DOPC = 5:2:2:1 (for Lynx1, Lynx2, Lypd6, Lypd6B, Ly6H)
CHL/POPC/NSM/POPE = 9:5:5:1 (for CD59)

Na^+ and Cl^- ions were used to neutralize the protein and to approximate physiological solution with [NaCl]=150 mM.

Table S3. Predicted amino acid pK_a values for Ly6 proteins.

Lynx1		Lynx2		Lypd6		Lypd6B		Ly6H		CD59	
NT	7.71	NT	7.83	NT	7.82	NT	7.99	NT	7.96	NT	7.92
D22	3.33	D36	2.99	D27	3.90	D52	3.95	D30	3.33	D37	3.16
D31	4.00	D52	2.35	D32	2.23	D70	3.69	D49	3.75	D47	3.36
D74	1.66	E29	4.57	D57	3.49	D80	3.89	D59	2.44	D49	1.91
D89	2.90	E30	4.90	D67	1.43	D125	3.93	D66	3.97	D74	2.17
E70	4.00	E41	4.27	D109	3.88	E66	4.53	D78	3.64	D92	3.05
H24	5.72	E57	3.76	D137	3.82	E79	3.47	D87	3.92	E68	4.38
H79	6.84	E60	4.13	E53	4.46	E114	2.11	D101	2.90	E81	4.09
Y28	10.15	Y26	10.46	E60	4.17	E127	3.71	D103	3.84	E83	4.59
Y45	10.05	Y67	10.97	E73	4.04	E130	2.96	D108	3.08	E98	4.74
Y52	10.09	Y84	10.25	E84	3.30	E136	3.95	E106	4.62	E101	4.49
Y53	10.07	K56	9.69	E100	4.11	E143	4.67	H39	6.28	H69	7.05
Y73	9.87	K69	8.56	E101	3.24	H94	5.09	H67	6.09	Y29	10.34
Y76	10.03	K92	10.39	E111	4.66	H95	5.76	H83	6.27	Y61	11.92
Y88	10.20	K112	10.46	E113	3.85	H99	6.39	Y88	10.11	Y86	10.41
K59	10.60	K113	10.44	E123	0.96	H116	6.81	K43	11.52	Y87	10.29
K62	9.44	R68	14.37	E135	4.11	H121	6.43	K65	10.46	K39	11.40
K78	10.45	R110	13.48	H37	7.00	H122	6.78	K71	9.17	K55	10.45
R38	13.53	R114	12.47	H81	4.74	H128	6.18	K81	10.51	K63	9.51
R50	12.45			H112	6.40	H148	6.24	K99	11.42	K66	10.62
R57	12.43			H115	6.98	H157	6.64	K107	10.46	K90	11.26
R67	12.57			Y35	10.21	Y45	10.08	R56	12.58	K91	10.48
				Y43	10.77	Y72	10.17	R64	12.45	R78	12.35
				Y59	8.81	Y89	10.30	R82	12.53	R80	12.44
				Y69	9.53	K40	10.46				
				Y76	10.15	K61	8.98				
				Y78	10.03	K81	11.37				
				K31	10.48	K107	7.30				
				K48	11.36	K108	11.23				
				K54	10.57	R48	12.29				
				K94	6.76	R76	12.19				
				K116	10.45	R101	12.38				
				R26	13.71	R112	12.53				
				R63	13.14	R124	12.38				
				R72	12.40	R132	13.57				
				R75	13.62	R160	12.42				
				R95	13.88						
				R108	12.40						
				R133	12.16						

Results of prediction are shown in two sub columns for each Ly6 protein: residue and pK_a value (as predicted by the PROPKA 3.4 program [3,4]) The numbering of residues is given in accordance with Uniprot. pK_a values for cysteine residues, forming disulfide bridges, are not shown. NT denotes N-termini amino groups.

Supplementary references

1 Procter JB, Carstairs GM, Soares B, Mourão K, Ofoegbu TC, Barton D, Lui L, Menard A, Sherstnev N, Roldan-Martinez D, Duce S, Martin DMA & Barton GJ (2021) Alignment of Biological Sequences with Jalview. *Methods Mol Biol* **2231**, 203–224.

2 Shafee TMA, Robinson AJ, van der Weerden N & Anderson MA (2016) Structural homology guided alignment of cysteine rich proteins. *Springerplus* **5**, 27.

3 Søndergaard CR, Olsson MHM, Rostkowski M & Jensen JH (2011) Improved treatment of ligands and coupling effects in empirical calculation and rationalization of pKa values. *J Chem Theory Comput* **7**, 2284–2295.

4 Olsson MHM, Søndergaard CR, Rostkowski M & Jensen JH (2011) PROPKA3: Consistent treatment of internal and surface residues in empirical pKa predictions. *J Chem Theory Comput* **7**, 525–537.

Supplementary figures

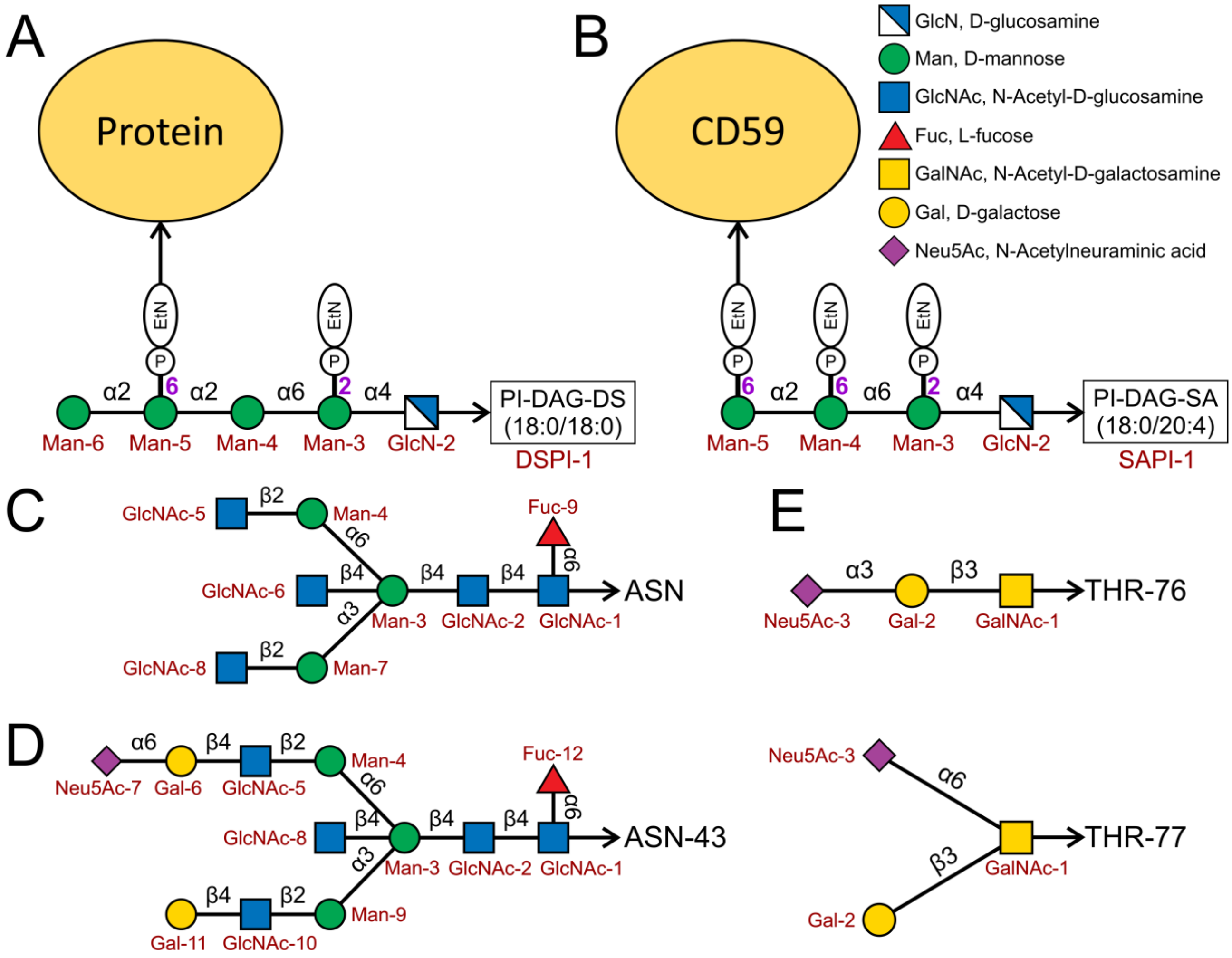

Figure S1. Used structures of GPI-anchors and glycans. A. GPI-anchor architecture used for Lynx1, Lynx2, Lypd6, Lypd6B and Ly6H models. **B.** CD59 GPI-anchor architecture. **C.** N-glycan composition used for Lynx2, Lypd6, Lypd6B and Ly6H models. **D.** CD59 N-glycan composition attached to Asn-43. **E.** CD59 O-glycans attached to Thr-76 and -77 residues. Glycosylation sites are shown in Table 1. Used abbreviations: PI — phosphatidylinositol, DAG — diacylglycerol, DS — distearoyl, DSPI — 1,2-distearoylphosphatidylinositol, SA — 1-stearoyl-2-arachidonoyl, SAPI — 1-stearoyl-2-arachidonoylphosphatidylinositol, P — phosphate, EtN — ethanolamine.

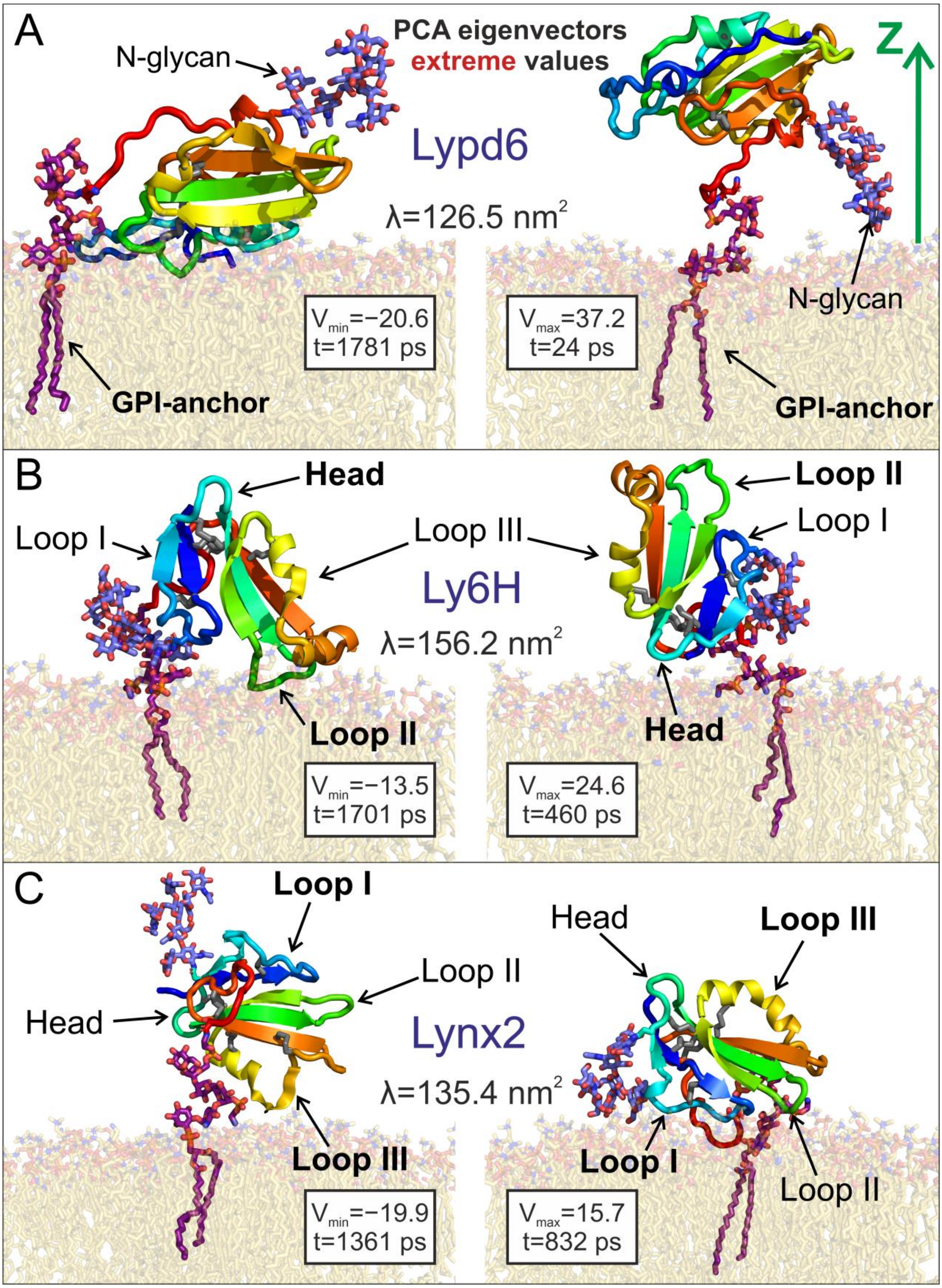

Figure S2. Extremal configurations revealed *via* principal component analysis (PCA). Configurations corresponding to minimal and maximal values of the first principal component eigenvector are shown for Lypd6 **(A)**, Ly6H **(B)** and Lynx2 **(C)**. Found PCA eigenvectors are able to characterize: elevation of a protein above the membrane **(A)**; head and loop II tilt, position and orientation **(B)**; loop I and loop III rotation towards the membrane **(C)**. λ denotes obtained eigenvalues; V_{min} and V_{max} — minimal and maximal eigenvectors values, respectively; t — MD time when eigenvectors reach extreme value. Revealed principal components provide a basis to introduce variables triad {Z, α, β} for more thorough systems examination (see Figures 1-3, S3-S5).

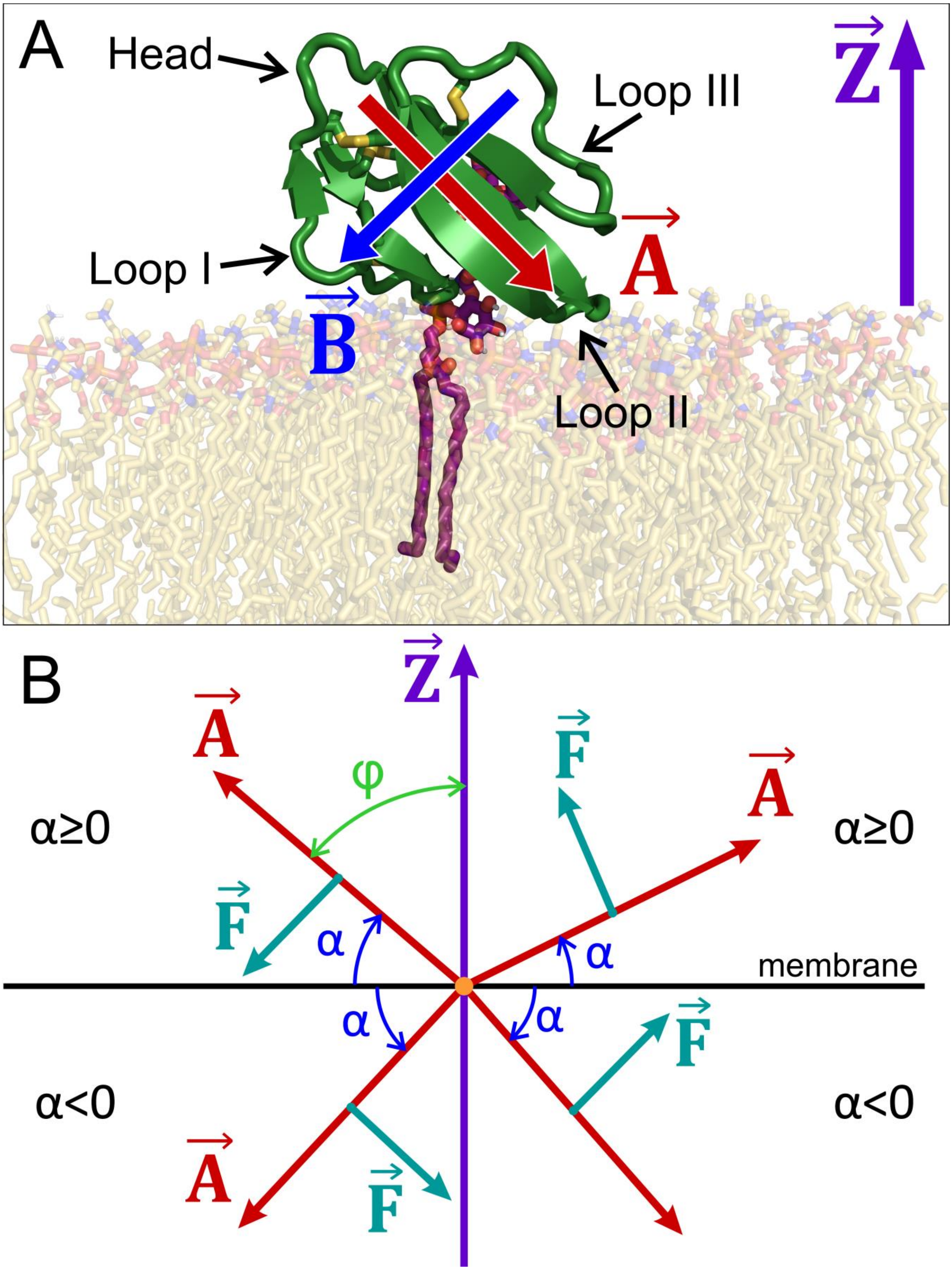

Figure S3. Tilt angle α definition. A. Schematic view of Lynx1 with orientation vectors $\vec{A}$ (connects 'head' and the tip of the loop II) and $\vec{B}$ (connects loop III and loop I, and orthogonal to $\vec{A}$). $\vec{Z}$ is the membrane normal. **B.** The definition of tilt angle (α) *via* angle $\varphi=\angle(\vec{A}, \vec{Z})$. Tilt angle represents the angle between $\vec{A}$ and membrane plane: $\alpha = 90° - \varphi$.

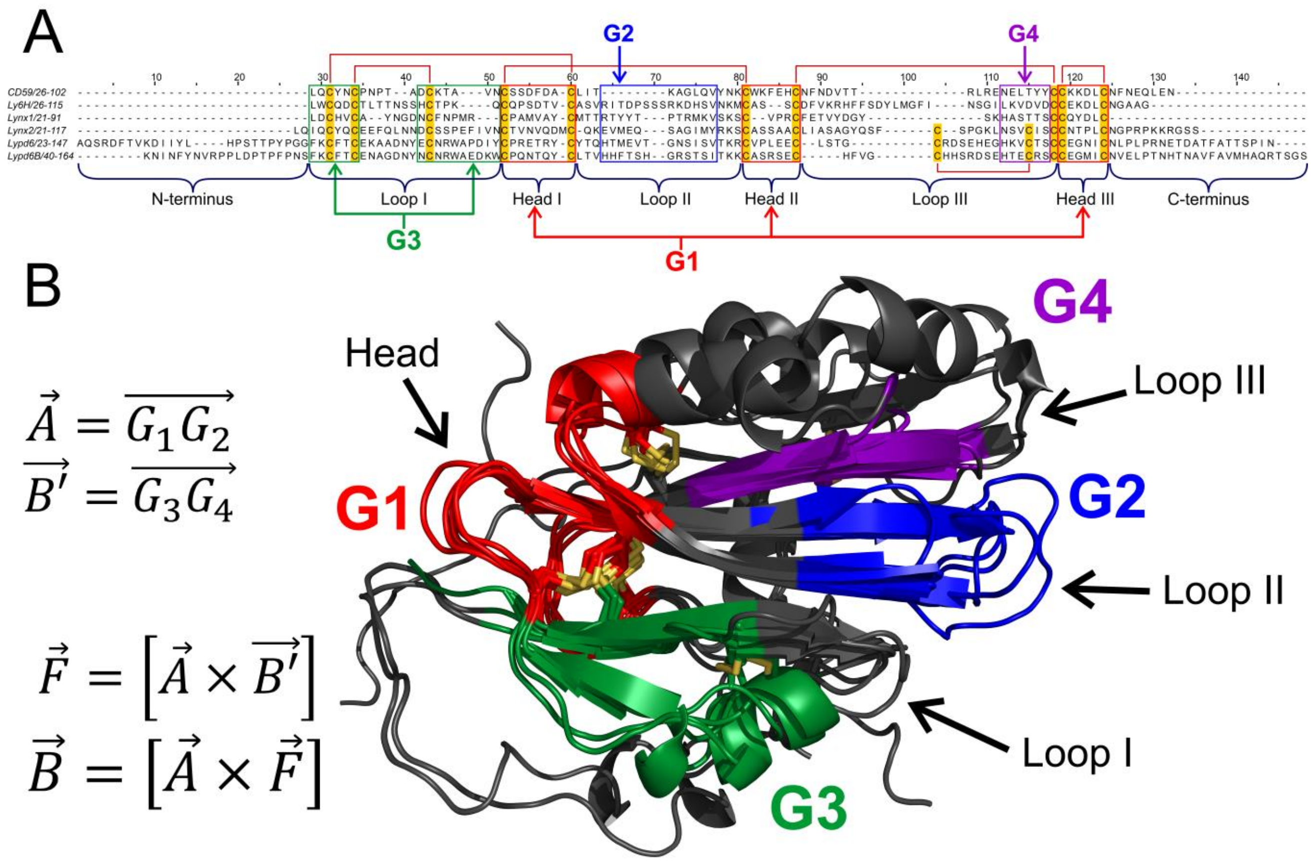

Figure S4. Groups selection for determination of protein orientations: alignment (**A**) and superposition (**B**). To set up two reference orientation vectors, four groups of amino acid residues (G1, G2, G3, and G4) were selected. We aimed to get two mutually perpendicular vectors $\vec{A}$ and $\vec{B}$, describing orientation of Ly6 proteins in 3D space (see Fig. S3). $\vec{A}$ connects groups G1 and G2; $\overrightarrow{B'}$ connects G3 and G4. In general, $\vec{A}$ and $\overrightarrow{B'}$ are not perpendicular. Using vector product $\vec{F}$, we find $\vec{B}$, which is orthogonal to $\vec{A}$ (see Fig. S5). $\vec{A}$ is utilized for tilt angle α definition (Fig. S3), $\vec{B}$ and $\vec{F}$ — for rotation angle β (Fig. S5).

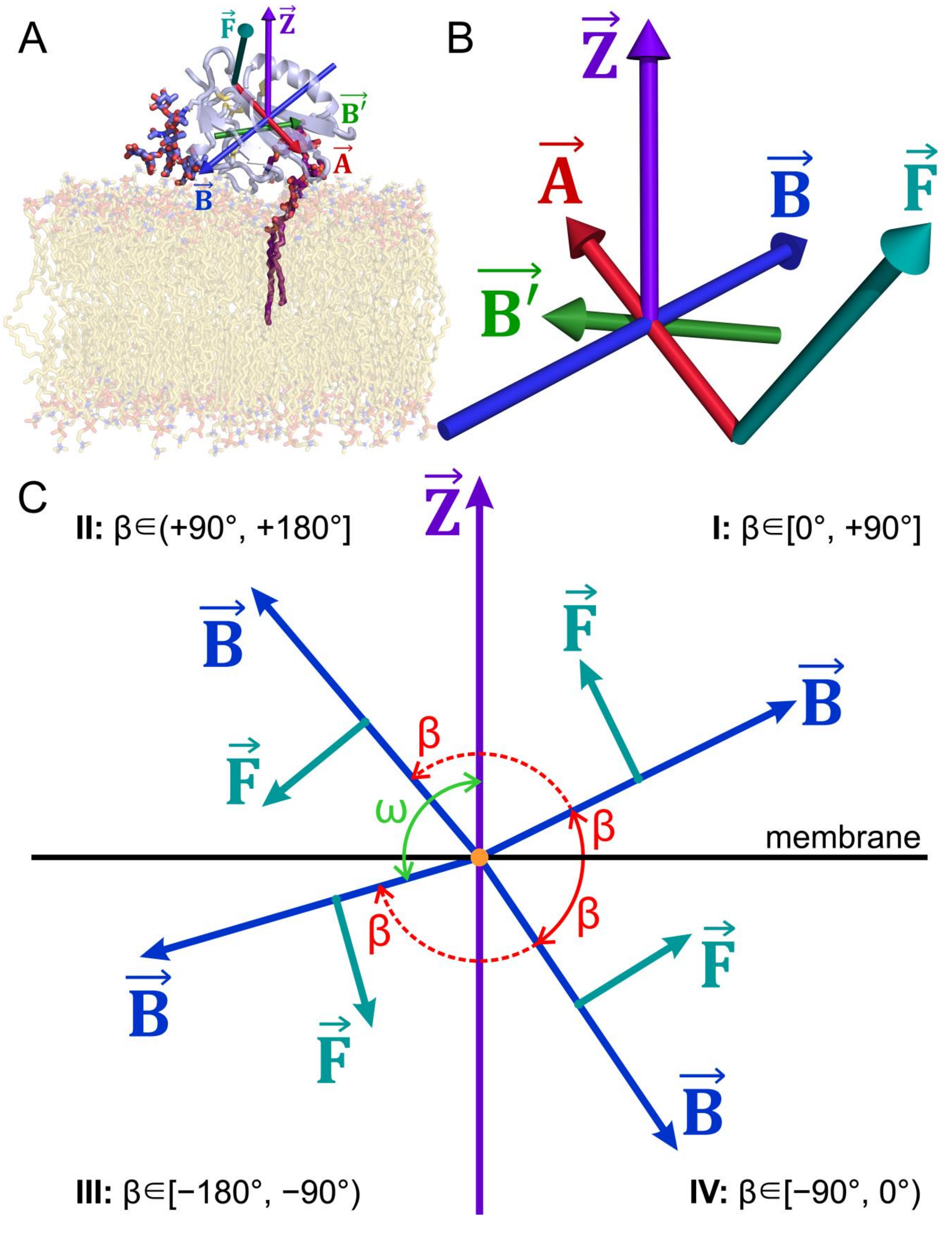

Figure S5. Vectors used for determination of proteins orientation (A,B) and rotation angle β definition (C). Vectors $\overrightarrow{A}$, $\overrightarrow{B'}$, $\overrightarrow{B}$, $\overrightarrow{F}$, and $\overrightarrow{Z}$ are shown on the Lynx2 molecule during MD **(A)** and separately **(B)**. **C.** Rotation angle β (β ∈ [−180°, +180°]) is defined differently in distinct quadrants I-IV *via* angle $\omega = \angle(\vec{B}, \vec{Z})$.

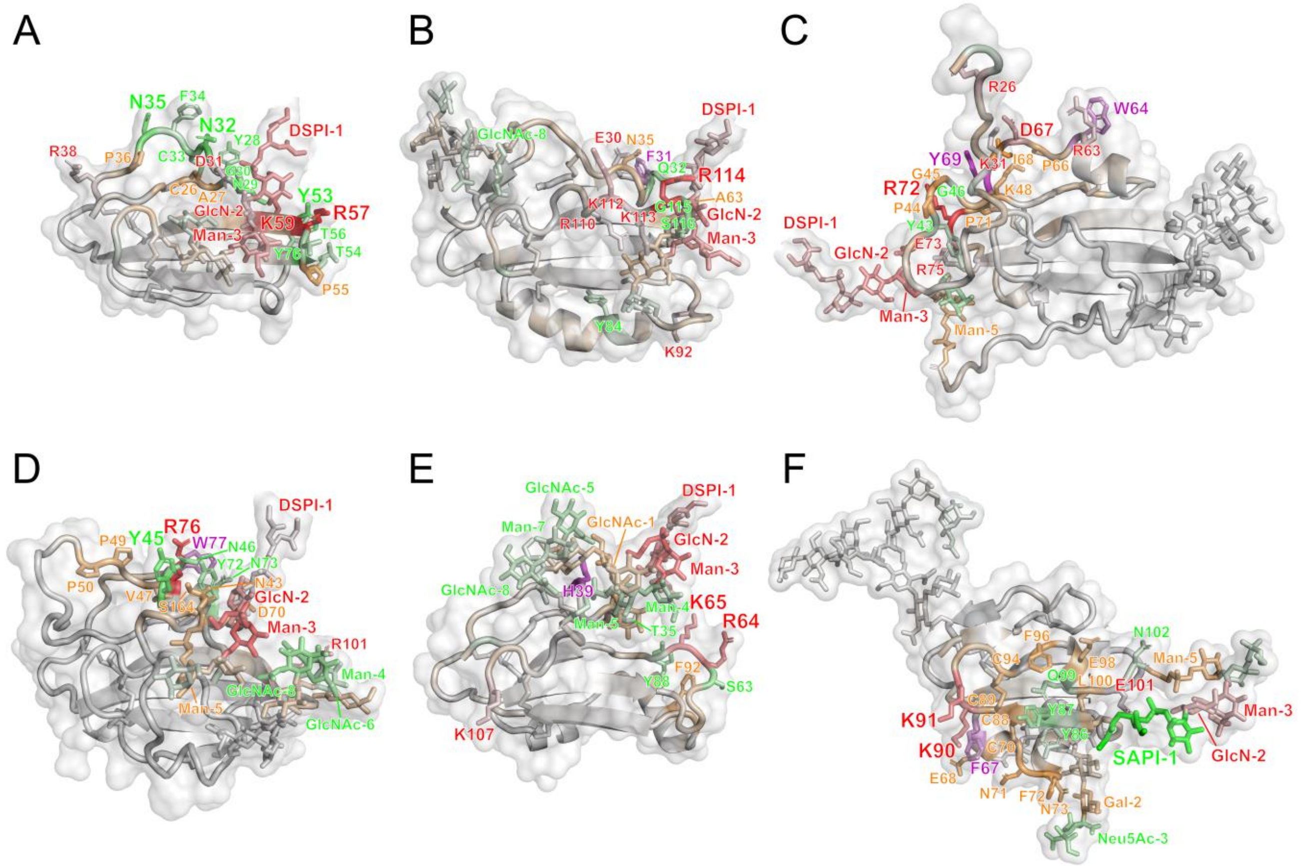

Figure S6. Membrane active residues of Lynx1 **(A)**, Lynx2 **(B)**, Lypd6 **(C)**, Lypd6B **(D)**, Ly6H **(E)**, CD59 **(F)**. Residues are colored according to predominant interaction type and relative lifetime in color spectra from *light gray* to: ***red*** (ion and ion-dipole interactions), ***green*** (hydrogen bonds), ***purple*** (π-cation interactions), ***orange*** (hydrophobic contacts). Most important residues (see Table 2) forming contacts with high relative lifetimes are signed.

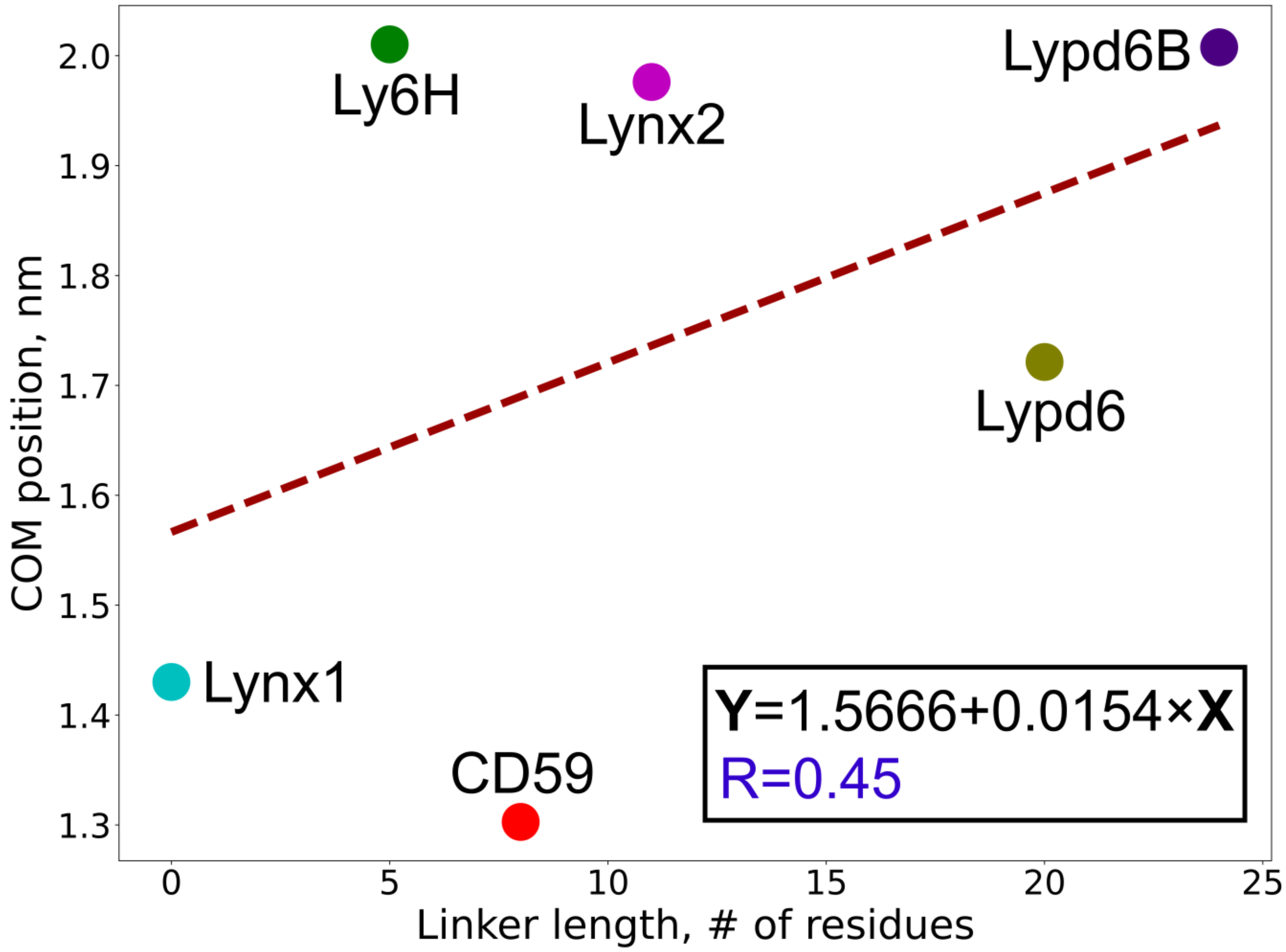

Figure S7. Absence of strong correlation between length of C-terminal linker in the proteins and COM positions. Correlation coefficient R=0.45 and fitted linear dependence are provided in the inset.

Supplementary movies and MD trajectories currently are available on Yandex Disk (https://disk.yandex.ru/d/XhgHbwwaOK_zMQ) and would be uploaded to the Zenodo repository upon the acceptance.
